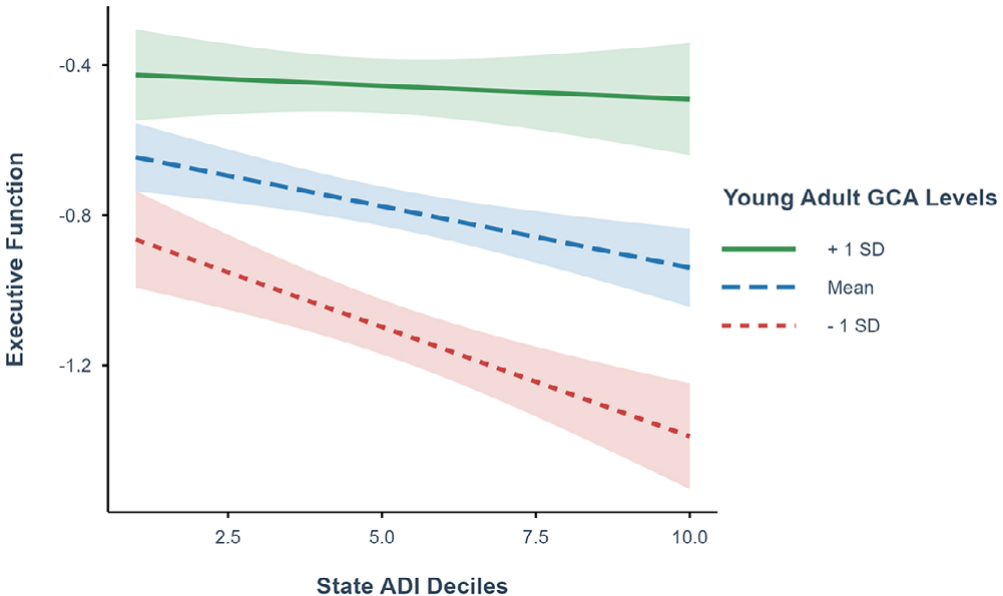# Young Adult Cognitive Reserve Moderates Neighborhood Disadvantage Effects on Later Life Executive Function: A Nationwide U.S. Study

**DOI:** 10.1002/alz70860_100563

**Published:** 2025-12-23

**Authors:** Rongxiang Tang, Jeremy A. Elman, Jack F.V. Hunt, Tyler R. Bell, Matthew S. Panizzon, William S. Kremen, Carol E. Franz, the Vietnam Era Twin Study of Aging coinvestigators

**Affiliations:** ^1^ Texas A&M University, College Station, TX, USA; ^2^ University of California San Diego, La Jolla, CA, USA; ^3^ University of California, San Diego, La Jolla, CA, USA; ^4^ Center for Behavior Genetics of Aging, University of California, San Diego, La Jolla, CA, USA

## Abstract

**Background:**

Neighborhood disadvantage captures the socioeconomic context of an individual's environment and is associated with poorer cognition and increased vulnerability to MCI and AD independently of individual‐level socioeconomic status (SES). Studies of neighborhood disadvantage and cognition in older adults are often constrained to populations from a single state or city, but are rarely conducted across the national level with a representative distribution of neighborhood disadvantage. Moreover, little is known about potential moderators of the relationship between neighborhood disadvantage and cognitive function. In particular, whether individual differences in prior level of cognitive reserve may attenuate the negative association between neighborhood disadvantage and different cognitive domains in older adulthood remains unexplored.

**Methods:**

In 1149 community‐dwelling dementia‐free men (mean age: 67.56; range: 61.37‐73.25) living across the US, we quantified each individual's neighborhood level of socioeconomic disadvantage using the area deprivation index (ADI), a validated index based on American Community Survey data. We assessed associations between ADI and five cognitive domains (executive function, episodic memory, processing speed, verbal fluency, visual‐spatial ability), while controlling for individual differences in young adult cognitive reserve (i.e., general cognitive ability [GCA] assessed on average at the age of 20) and household SES. We then investigated the moderating effect of cognitive reserve on the ADI‐cognition associations. As a comparison, we also examined years of education as a moderator of the ADI‐cognition associations.

**Results:**

Greater neighborhood disadvantage was associated with poorer executive function (β=‐0.09, *p* < .05) and processing speed (β=‐0.12, *p* < .05), but not episodic memory, visual‐spatial ability or verbal fluency. Moreover, the negative impact of neighborhood disadvantage on executive function was weaker among individuals with greater young adulthood cognitive reserve (ADI x GCA: β=0.07, *p* < .05) but not more years of education.

**Conclusions:**

Higher young adulthood cognitive reserve confers greater cognitive resilience against neighborhood disadvantage in older adulthood. Education did not show the same effect. Fostering early life cognitive development to enhance cognitive reserve—perhaps through improved nutrition and education quality—may buffer against environmental threats to executive function, which is among the earliest cognitive functions affected in later life. That, in turn, may decrease individual vulnerability to MCI and AD.